# European maize landraces made accessible for plant breeding and genome-based studies

**DOI:** 10.1007/s00122-019-03428-8

**Published:** 2019-09-26

**Authors:** Armin C. Hölker, Manfred Mayer, Thomas Presterl, Therese Bolduan, Eva Bauer, Bernardo Ordas, Pedro C. Brauner, Milena Ouzunova, Albrecht E. Melchinger, Chris-Carolin Schön

**Affiliations:** 1 Plant Breeding, TUM School of Life Sciences Weihenstephan, Technical University of Munich, 85354 Freising, Germany; 2 Maize Breeding, KWS SAAT SE, 37574 Einbeck, Germany; 3 Misión Biológica de Galicia, Spanish National Research Council (CSIC), 36080 Pontevedra, Spain; 4 Institute of Plant Breeding, Seed Science and Population Genetics, University of Hohenheim, 70593 Stuttgart, Germany; 5 Present Address: Maize Breeding, KWS SAAT SE, 37574 Einbeck, Germany

## Abstract

**Key message:**

Doubled-haploid libraries from landraces capture native genetic diversity for a multitude of quantitative traits and make it accessible for breeding and genome-based studies.

**Abstract:**

Maize landraces comprise large allelic diversity. We created doubled-haploid (DH) libraries from three European flint maize landraces and characterized them with respect to their molecular diversity, population structure, trait means, variances, and trait correlations. In total, 899 DH lines were evaluated using high-quality genotypic and multi-environment phenotypic data from up to 11 environments. The DH lines covered 95% of the molecular variation present in 35 landraces of an earlier study and represent the original three landrace populations in an unbiased manner. A comprehensive analysis of the target trait plant development at early growth stages as well as other important agronomic traits revealed large genetic variation for line per se and testcross performance. The majority of the 378 DH lines evaluated as testcrosses outperformed the commercial hybrids for early development. For total biomass yield, we observed a yield gap of 15% between mean testcross yield of the commercial hybrids and mean testcross yield of the DH lines. The DH lines also exhibited genetic variation for undesirable traits like root lodging and tillering, but correlations with target traits early development and yield were low or nonsignificant. The presented diversity atlas is a valuable, publicly available resource for genome-based studies to identify novel trait variation and evaluate the prospects of genomic prediction in landrace-derived material.

**Electronic supplementary material:**

The online version of this article (10.1007/s00122-019-03428-8) contains supplementary material, which is available to authorized users.

## Introduction

Maize (*Zea mays* L. ssp. *mays*) seed banks around the world harbor thousands of landrace accessions, representing a rich resource of currently untapped native diversity that could be harnessed for plant improvement and adaptation to environmental changes (Hoisington et al. 1999; Ortiz et al. 2010; McCouch et al. 2013; Hellin et al. 2014; Wang et al. 2017). European flint maize went through several bottlenecks, the first of which occurred in the Americas (Doebley et al. 1986), followed by the introduction to Europe (Rebourg et al. 2003). In the course of maize breeding, landraces were replaced by hybrids. For the establishment of hybrid breeding, only a limited set of founder landraces was sampled, and the inbred lines produced were subjected to second cycle breeding (Messmer et al. 1992; Barrière et al. 2006). Subsequent selection at high intensities has led to an additional decline in genetic diversity of elite germplasm, especially within the flint heterotic pool important for European maize breeding (Messmer et al. 1992; Reif et al. 2005a, b; Lu et al. 2009). Revisiting the vast diversity of landraces stored in seed banks is considered a promising approach for broadening the genetic base of current germplasm pools (Pollak 2003; Salhuana and Pollak 2006; Warburton et al. 2008; Strigens et al. 2013; McCouch et al. 2013; Navarro et al. 2017). However, opening this avenue for quantitative traits entails considerable challenges, and efficient strategies are still lacking.

In a first step, the most promising landraces have to be identified from several thousand stored in seed banks, even if only the flint pool is of interest. Information on stored landraces is limited, and the choice has to be based either on passport data from seed banks, or the per se and/or testcross performance of the landraces has to be evaluated in field trials (Pollak 2003; Salhuana and Pollak 2006; Böhm et al. 2014). In allogamous species like maize, landrace collections represent populations of heterogeneous and heterozygous individuals. Thus, the evaluation of populations either per se or in testcrosses would disregard the large genetic variation found within landraces, and without prior self or cross, it is not possible to evaluate the breeding potential of individual genotypes. In order to harness the genetic diversity within landraces, reproducible genetic units such as libraries of doubled-haploid (DH) lines from landraces have been suggested to overcome some of the aforementioned drawbacks since they are suitable for genotyping and high-precision phenotyping (Wilde et al. 2010; Strigens et al. 2013; Melchinger et al. 2017). Diversity from landraces captured in such DH libraries could help in improving traits such as plant development at early growth stages, for which genetic variation is small in breeding material. However, improving quantitative traits by utilizing lines derived from landraces is complex because the targeted introgression of favorable alleles at major genes is not possible (Bernardo 2002). Any introgression of landrace material therefore carries the risk of an undesired correlated response in traits other than the trait under selection due to the overall poor agronomic performance of the landrace material. To achieve a targeted utilization of natural diversity, an exhaustive characterization of line per se and testcross performance for the trait of interest and as many other agronomic and morphological traits as possible has to be carried out in order to develop a pre-breeding strategy that allows introgression of favorable diversity into elite germplasm without introducing major disadvantages in other traits (Sood et al. 2014).

In the research at hand, we employed large-scale production of DH lines to make native diversity for quantitative traits in maize landraces accessible for the purpose of germplasm improvement and genome-based studies. Our objectives were (i) to create a publicly available diversity atlas of European flint maize by characterizing landrace-derived DH libraries genotypically and phenotypically for line per se and testcross performance, (ii) to provide a comprehensive analysis of the DH libraries in terms of population structure, performance level, trait correlations, and genetic variances for a broad range of traits, and (iii) to gain insights into potential strategies for capturing native diversity for use in germplasm improvement.

## Materials and methods

### Plant material

The three landraces Kemater Landmais Gelb (KE, Austria), Petkuser Ferdinand Rot (PE, Germany), and Lalin (LL, Spain) were chosen for the production of DH lines because they showed phenotypic variation for early development as well as low levels of linkage disequilibrium (LD) and population structure within populations. They were selected from a set of 35 European maize landraces covering a broad geographical region of Europe that was described in detail by Mayer et al. (2017). Together, they represented 95.0% of the molecular variance of the full set of 35 landraces. From the selected landraces, 1015 DH lines (516 KE, 432 PE, 67 LL) were produced and multiplied using the in vivo haploid induction method (Röber et al. 2005). Phenotyping of lines per se (LP) was conducted in 2017 and 2018. Testcrosses (TC) of a subset of 378 DH lines from landraces KE and PE were evaluated in 2018. To warrant successful TC evaluation, the shortest, earliest, and late maturing lines as well as lines with a high score for lodging were not included in the TC production. The dent line F353 (Institut national de la recherche agronomique, INRA) was used as the female parent in TC production to ensure uniform seed quality across DH lines and because variation in tassel architecture of DH lines hampered detasseling.

### Analysis of genotypic data and population structure

The 1015 DH lines and 144 S_0_ plants (48 per landrace) from the landraces KE, PE, and LL were genotyped using the 600 k Affymetrix^®^ Axiom^®^ Maize Array (Unterseer et al. 2014). Only markers assigned to the best quality class (Unterseer et al. 2014), with a call rate of ≥ 0.9 and with a known physical position in the B73 reference sequence [AGPv4, (Jiao et al. 2017)], were used for the analyses. One S_0_ plant from landrace PE was excluded due to an insufficient call rate (≤ 0.9). Assignment of lines to their respective landrace was performed using the ADMIXTURE software tool (Alexander et al. 2009) in supervised mode with three pre-defined groups (KE, PE, and LL) that were determined from S_0_ plants. DH lines with less than 75% concordance with the landrace to which they were assigned by pedigree records were excluded from further analysis. Markers and individuals with > 10% missing values were removed. In DH lines, markers and individuals with > 5% heterozygous genotype calls were discarded, and all remaining heterozygous calls were set to missing values. Missing values in the DH lines were imputed separately for each landrace using BEAGLE 5.0 (default parameters) (Browning et al. 2018). Missing values in the S_0_ plants were imputed, and two gametes were phased from each S_0_ plant separately in each landrace using BEAGLE 5.0 (iterations = 50, phase-segment = 10, phase-states = 500) and a reference panel consisting of the corresponding DH lines. Pairwise modified Rogers’ distances [MRD; (Wright 1978)] were calculated, and DH lines showing a pairwise MRD of < 0.05 were assumed to be duplicates and excluded from further analyses. Markers were identified which overlapped between DH lines and S_0_ gametes. Quality filtering and imputation resulted in 941 DH lines (501 KE, 409 PE, and 31 LL) and 286 S_0_ gametes (96 KE, 94 PE, and 96 LL) genotyped with 499,574 common markers.

We performed a principal coordinate analysis [Gower (1966), R-package ape] based on MRD for DH lines and S_0_ plants. The MRD matrices of DH lines and S_0_ plants were hierarchically clustered using the unweighted pair group method with arithmetic mean (UPGMA) implemented in the hclust function in R and are displayed as 1-MRD. In order to estimate the proportion of molecular variance explained by the three landraces under study, an analysis of molecular variance [AMOVA; Excoffier et al. (1992)] was performed to partition the molecular variation into within- and between-landrace components. This analysis used the panel of 35 European landraces described by Mayer et al. (2017) for comparison. In addition, a second AMOVA decomposing the variance within and between DH lines and S_0_ gametes was performed to investigate how much of the molecular variance lies within and between those two groups.

### Field experiments and phenotypic analysis

Line per se (LP) performance was evaluated in Germany during 2017 using ten separate 10 × 10 lattice designs in four locations (1000 entries: 958 DH lines plus checks) and during 2018 using eight 10 × 10 lattice designs in three locations (800 entries: 756 DH lines plus checks). A randomly chosen subset (five 10 × 10 lattice designs, 458 and 468 DH lines plus checks in 2017 and 2018, respectively) was evaluated in two locations in Spain in both years. The trial locations were Einbeck (EIN, Germany, 2017 + 2018), Roggenstein (ROG, Germany, 2017 + 2018), Bernburg (BBG, Germany, 2017), Klein Wanzleben (KLW, Germany, 2018), Oberer Lindenhof (OLI, Germany, 2017), Golada (GOL, Spain, 2017 + 2018), and Tomeza (TOM, Spain, 2017 + 2018). See Table S1 for a detailed description of the test locations [geographical coordinates, elevation, precipitation, temperature; the climate data was obtained from the Bavarian State Research Center for Agriculture, Landwirtschaftliches Technologiezentrum Augustenberg, and Menne et al. (2012)]. Each combination of year and location was considered to be one environment in later analyses. The number of lines tested had to be reduced between 2017 and 2018 due to seed shortage and the exclusion of lines that did not pass the quality control described above for the genotypic data analysis. In 2017, 14 flint (CH10 provided by Agroscope Changins-Wädenswil (Switzerland); D152, DK105, UH006, UH007, and UH009 provided by the University of Hohenheim (Germany); EP1 and EP44 provided by Misión Biológica de Galicia, Consejo Superior de Investigaciones Científicas, (CSIC, Spain); F03802, F2, F283, F64, and F7 provided by Institut national de la recherche agronomique (INRA, France); EC49A provided by Centro de Investigaciones Agrarias Mabegondo, Instituto Galego da Calidade Aumentaria (CIAM-INGACAL, Spain) and one dent (F353, INRA, tester in testcross evaluation) inbred line served as checks and were included as duplicate entries. The checks were chosen in order to exhibit variation in plant development at early growth stages and flowering time. In 2018, the number of checks was reduced to four lines (DK105, EP1, F2, and F353) included in each lattice design per location (eight in Germany, five in Spain). In both years, the three landraces were included as quadruplicate entries. Plots were single rows 3 m in length with a distance of 0.75 m between rows and twenty plants per plot, corresponding to a sowing density of about 9 plants m^−2^.

The testcrosses (TC) were evaluated in four 10 × 10 lattice designs in four locations in Germany in 2018 (EIN, KLW, ROG, OLI). In the TC trials, testcrosses of lines DK105, EP1, and F2 as well as testcrosses of the two landraces KE and PE and two commercial hybrid varieties (CH1 = KWS Stabil, CH2 = KWS Figaro) were planted as checks. The testcrosses of landraces KE and PE were planted in one lattice only, while all other checks were planted in every lattice. In TC, plots were double rows 5 m in length at locations ROG and OLI and 6 m in length at locations KLW and EIN, in both cases with 0.75 m distance between rows. Sowing density followed local practice at the experimental stations and varied between 9 and 11 plants m^−2^. Fertilization and plant protection were carried out according to standard agricultural practices in both the LP and the TC trials.

In the LP trial, a total of 25 morphological, agronomic, and early-development-related traits were measured (Table S2 provides detailed information on trait × environment combinations). The traits that were scored in ≥ 10 environments included emergence (EME, ratio of emerged plants to sown seeds, %), early vigor (EV, at three different growth stages V3, V4, and V6, 1–9 score, 1 = very poor vigor, 9 = very vigorous), early plant height (PH, at V4 and V6, average over three measured plants per plot, cm), final plant height (PH_final, cm), and female flowering (FF, d). Root lodging at the R6 stage (RL, 1 = no lodging, 9 = all plants showing severe lodging) was scored in six environments; tillering (TILL, 1 = no tillers, 9 = all plants showing many and long tillers) and male flowering (MF, d) were scored in five environments. The anthesis-silking interval (ASI, d) was calculated for the environments in which both MF and FF were scored. Ear height (EH, cm) was measured in four environments. In the Spanish environments, physiological traits like the maximum efficiency of photosystem II [Fv/Fm, using a fluorometer (OS-30p, Opti-Sciences Inc., USA)] were measured at stages V4 (2017 + 2018) and V6 (only 2017), and leaf greenness (SPAD) was measured by a chlorophyll content meter (CCM-200, Opti-Sciences Inc., USA; V3, V4 in both years, V6 only 2017). Reaction to stress was scored as cold tolerance (CT, 1–9 score, 1 = low cold tolerance, 9 = high cold tolerance; symptoms were chlorosis and necrosis on the leaves) after a very cold night with a slight frost at OLI 2017, drought/heat tolerance (DT, 1–9 score, 1 = low drought/heat tolerance, 9 = high drought/heat tolerance; symptoms were dry leaves and tassels) at EIN 2018, and rust susceptibility (binary) at TOM 2018. Traits related to tassel architecture were measured in ROG 2018. Tassel length was measured from the lowest tassel branch to the tassel tip (TL, cm), spike length was measured as the length of the top spike (SL, cm), the number of branches was counted (NB), and the tassel angle was scored on a 1–9 scale (TA, 1 = completely upright, 9 = branches horizontal). In the TC trial, EME, EV, PH, EH, PH_final, FF, TILL, and RL were scored as was described for LP. In addition, TC plots were harvested with a forage harvester to measure total dry matter yield (TDMY, dt/ha) and dry matter content (DMC, through near infrared spectroscopy or drying, in %).

The statistical model for estimating genotype and genotype × environment interaction variance components for lines derived from the same landrace was

1 $$y_{ijkopst} = \mu + m_{i} + \delta_{ij} l_{j} + g_{{k\left( {ij} \right)}} + u_{o} + \delta_{ij} lu_{jo} + gu_{{ko\left( {ij} \right)}} + k_{p\left( o \right)} + r_{{s\left( {op} \right)}} + b_{{t\left( {ops} \right)}} + \varepsilon_{ijkopst}$$

where *i* = 1, 2, 3 denotes three groups, i.e., DH lines from landraces (DHL), checks (CH), and landrace populations (LR_S_0_); *j* = 1, 2, 3 denotes the three landraces KE, PE, and LL; *µ* is the overall mean; $m_{i}$ is the effect of group *i*; $l_{j}$ is the effect of landrace *j* in group *i* = 1; $\delta_{ij}$ is a dummy variable with $\delta_{ij}$ = 1 for *i* = 1 and *j* = 1, 2, 3 and $\delta_{ij}$  = 0 otherwise; $g_{{k\left( {ij} \right)}}$ is the effect of line *k* nested in group *i* and landrace *j*; $u_{o}$ is the effect of environment *o*; $lu_{jo}$ is the interaction of landrace *j* and environment *o*; $gu_{{ko\left( {ij} \right)}}$ is the interaction effect for genotype *k* and environment *o*. The effects $k_{p\left( o \right)}$, $r_{{s\left( {op} \right)}}$, $b_{{t\left( {ops} \right)}}$, and $\varepsilon_{ijkopst}$ refer to the effect of the lattice (nested in environments), replicate (nested in lattices in environments), incomplete block (nested in replicates in lattices in environments), and the residual error, respectively. All effects except $m_{i}$ and $l_{j}$ were treated as random. Genotype and genotype × environment ($gu_{{ko\left( {ij} \right)}}$) variance components were modeled individually for the three landraces (*j* = 1, 2, 3), assuming that DH lines across and within landraces were unrelated. Residuals were assumed to be normally distributed with mean zero and two heterogeneous variances, one for $\delta_{ij} = 1$ and one for $\delta_{ij} = 0$ assigning the same residual variance to all three landraces in all environments. Raw data and outliers were manually curated by inspection of residual plots. Since genotyping and the first year of phenotyping were carried out in parallel, some lines were evaluated in the field during 2017 that did not pass quality control in the genotypic data analysis. Measurements for those entries were treated as missing values in the data analysis. The same model was used for the analysis of TC experiments, except that *i* = 1, 2 referred to DHL and CH and *j* = 1, 2 referred to landraces KE and PE. Restricted maximum-likelihood estimation implemented in the ASReml-R package (Butler et al. 2009) was used for estimating variance components and their standard errors. Differences among means $l_{j}$ were tested with pairwise *t*-tests using the R-package asremlPlus. Trait heritabilities were calculated on an entry-mean basis within landraces (Hallauer et al. 2010), and standard errors of heritability estimates were derived from standard errors of corresponding variance components using the delta method (Holland et al. 2010). Heritabilities and variance component estimates exceeding twice their standard errors were considered significant. Best linear unbiased estimates (BLUEs) of the genotype mean for each trait and DH line were obtained from a simplified version of the model in Eq. (1), dropping factors $m_{i}$, $\delta_{ij} l_{j}$ and $\delta_{ij} lu_{jo}$ and treating genotype ($g_{k}$) as a fixed effect. This model was also used to form linear contrasts used to test for significant differences (*t*-tests) between original landraces and the mean of the corresponding DH library (LP and TC) and between the mean of the two check hybrids and the mean of the DH library (TC only). We calculated the predicted response from selection within DH libraries (LP and TC) according to Falconer and Mackay (1996) as $\Delta G_{\left( \alpha \right)} = i_{\left( \alpha \right)} h\sigma_{G}$, where $i_{\left( \alpha \right)} =$ selection intensity for selection with $\alpha = 10\% \left( {i_{{\left( {10\% } \right)}} \approx 1.76} \right)$, $h =$ square root of heritability, and $\sigma_{G} =$ genetic standard deviation. To account for mean differences and different selection responses, we calculated the usefulness criterion (Schnell 1983) as $U_{{\left( {10\% } \right)}} = \bar{x} \pm \Delta G_{{\left( {10\% } \right)}}$ where $\bar{x} =$ mean of the respective DH library. Phenotypic correlations among traits were calculated from BLUEs as Pearson correlation coefficients within libraries in LP and TC, respectively. For evaluating the prospects of selection on LP performance in this material, we calculated Spearman rank correlations for same traits across LP and TC. To adjust for multiple testing, Bonferroni–Holm correction was applied for significance tests of phenotypic correlations in each DH library (Holm 1979). For estimating genetic covariances and genetic correlations between traits, the model in Eq. (1) was expanded to a bivariate model with pairs of traits. Genetic correlations were considered significant if they exceeded twice their standard error. The same method was applied for estimating genetic correlations between LP and TC performance.

In summary, high-quality phenotypic line per se data are available from up to 11 environments for 899 DH lines (471 KE, 402 PE, and 26 LL) and for a subset of 378 lines (190 KE, 188 PE) that were evaluated as testcrosses in four environments. For all lines, data on almost 500,000 SNP markers are available.

## Results

### Population structure and molecular variation

**Fig. 1:**
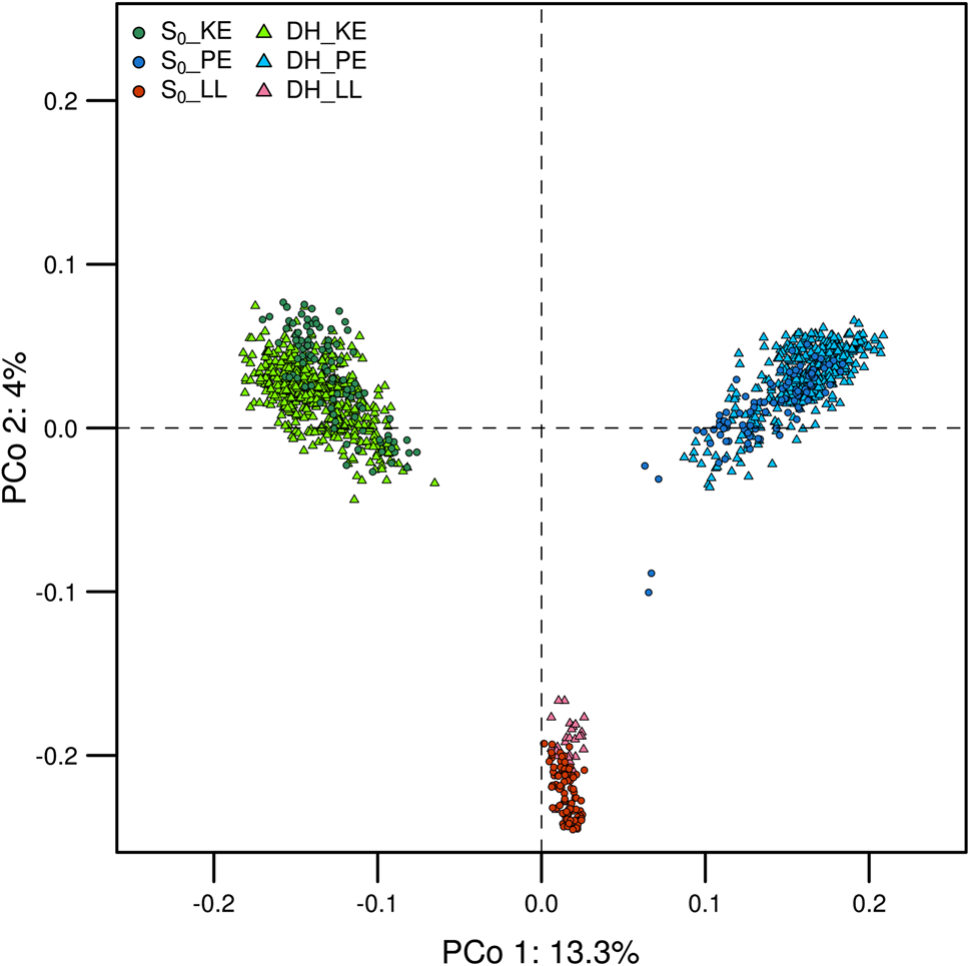
Principal coordinate analysis (PCoA) of DH libraries and S_0_ gametes based on modified Rogers’ distances between individuals. Landrace KE is colored in *green*, PE in *blue*, and LL in *red*. Darker colors were used for S_0_ gametes and brighter ones for DH. S_0_ gametes were plotted as *filled circles* and DH lines as *filled triangles*. Axis labels show the percentage of explained variance per principal coordinate (PCo)

**Fig. 2:**
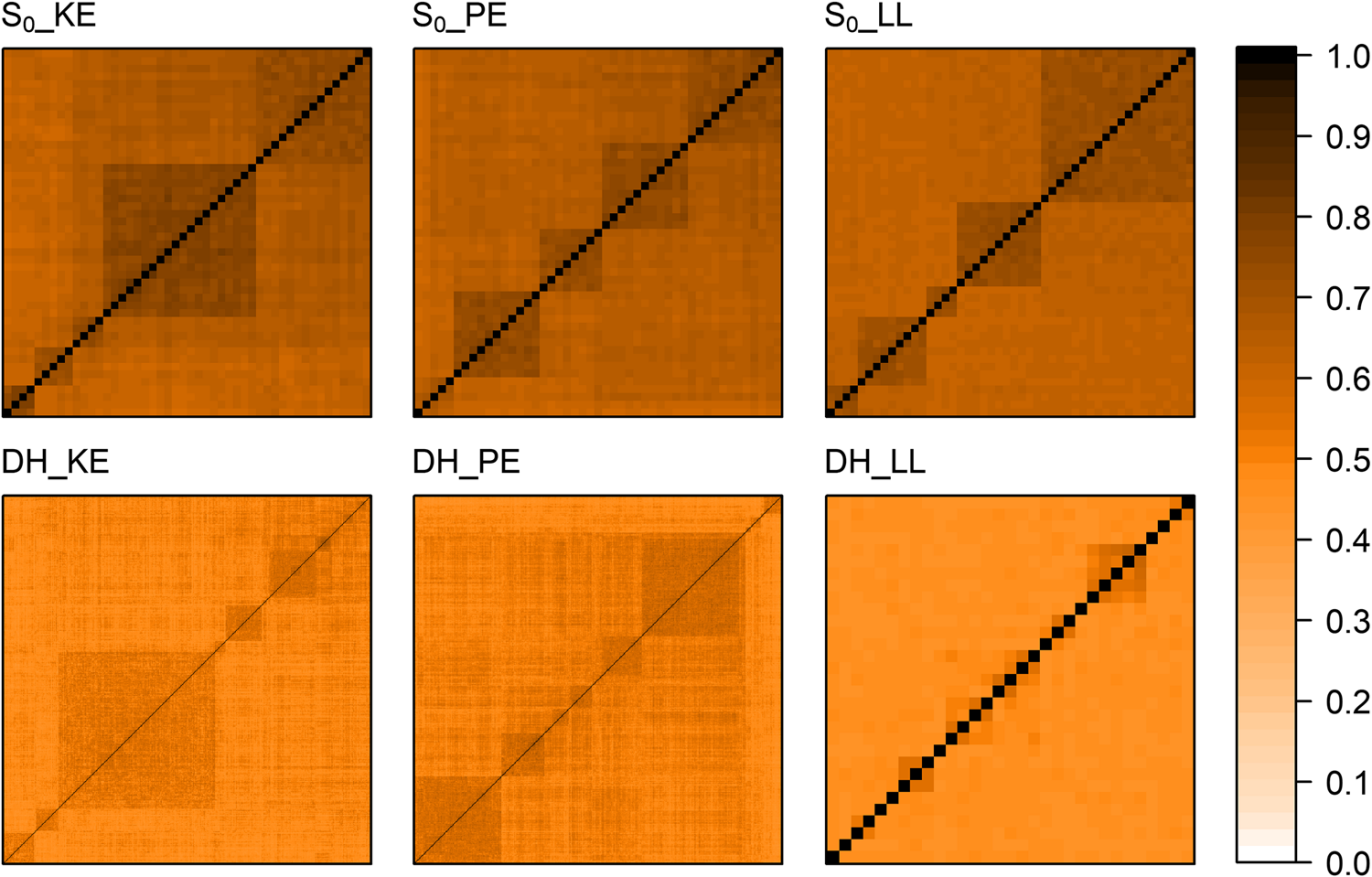
Heatmaps of 1-MRD matrices S_0__KE (*N* = 48 individuals), DH_KE (*N* = 471 lines), S_0__PE (*N* = 47 individuals), DH_PE (*N* = 409 lines), S_0__LL (*N* = 48 individuals), and DH_LL (*N* = 31 lines). Matrices were ordered according to hierarchical clustering with UPGMA

The principal coordinate analysis clearly separated the three landraces, with the first two coordinates explaining 13.3% and 4% of the total molecular variance, respectively (Fig. 1). DH lines and S_0_ gametes derived from the same landrace clustered together except for four gametes from S_0__PE, which fell outside the PE-cluster. Complementing our data with those from Mayer et al. (2017) revealed that S_0_ gametes sampled from landraces KE, PE, and LL individually accounted for 77, 75, and 89% of the total molecular variance captured in the collection of 35 European landraces used in their study. The AMOVA on S_0_ gametes and DH lines from the same landrace confirmed the results from the PCoA. While 95.3, 96.6, and 96.7% of the molecular variance were found within S_0_ and DH of KE, PE, and LL, respectively, less than 5% of the molecular variance was explained by differences between S_0_ gametes and DH lines of different landraces. Matrices of 1-MRD (Fig. 2) gave no indication of pronounced population structure for either DH or S_0_ plants. As expected, the similarity between S_0_ plants within landraces was on average higher than in DH lines due to the higher level of heterozygosity in the former.

### Phenotypic variation within and across landraces

**Fig. 3:**
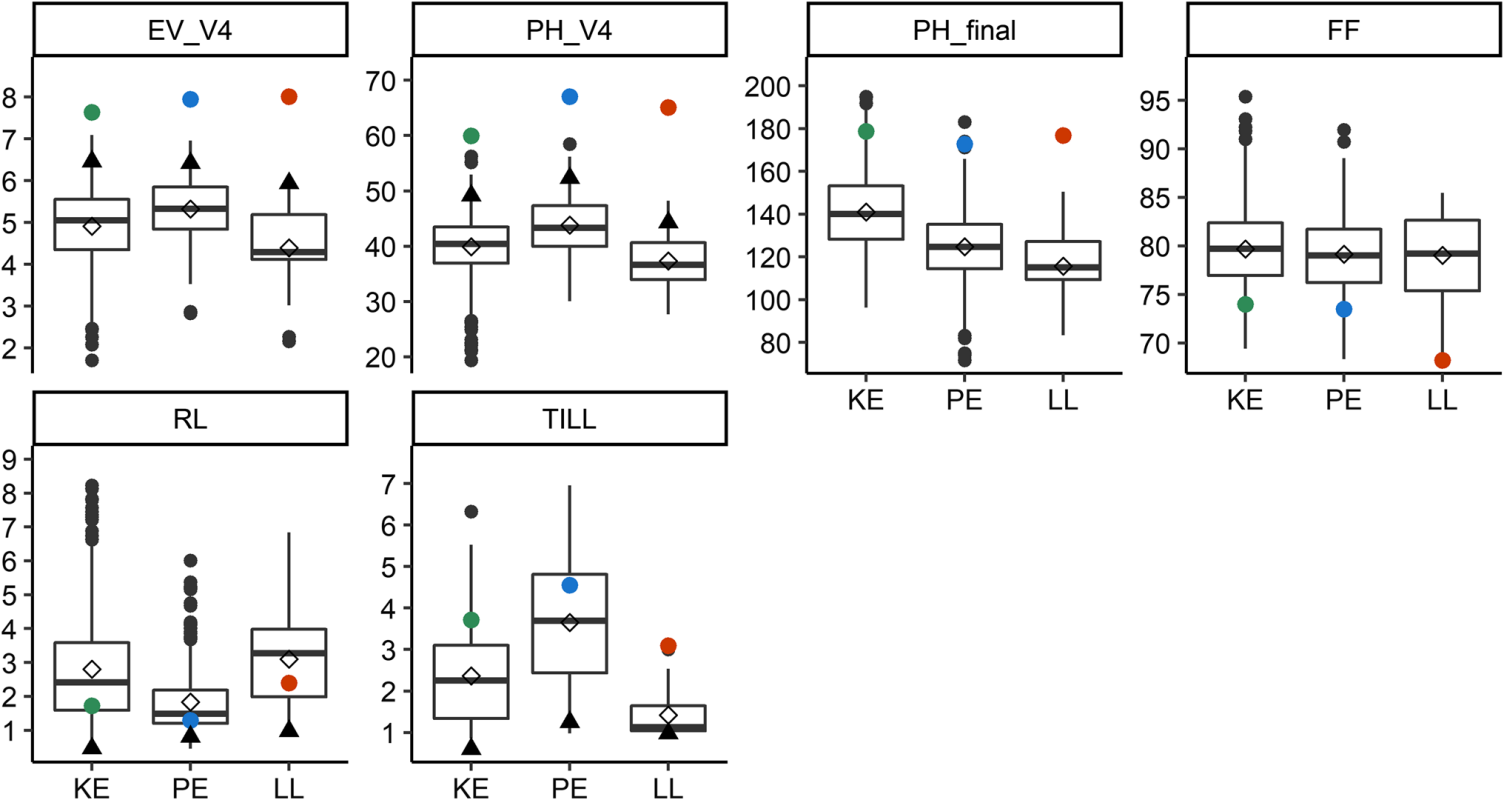
Boxplots of phenotypic data for line per se (LP) performance for the DH libraries from landraces KE, PE, and LL. Boxplots show the upper and lower quartiles, median (*horizontal bar*), mean (*open diamond*), whiskers (*vertical bars*), and the performance of the respective landrace (*filled circle* in *green*, *blue*, and *red* for KE, PE, and LL, respectively). Points above and below the whiskers indicate values ± 1.5 times the interquartile range. Usefulness for a selection intensity of 10% (U_10 %_) is indicated with *black filled triangles*. Traits are early vigor and early plant height at stage V4 (EV_V4, PH_V4), final plant height (PH_final), female flowering (FF), root lodging (RL), and tillering (TILL)

**Fig. 4:**
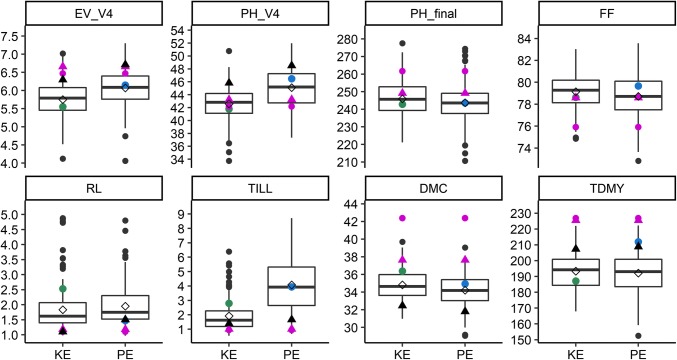
Boxplots of phenotypic data for testcross (TC) performance for DH libraries from landraces KE and PE. Boxplots show the upper and lower quartiles, median (*horizontal bar*), mean (*open diamond*), whiskers (*vertical bars*) and the performance of the respective landrace (*filled circle* in *green* and *blue* for KE and PE, respectively). Points above and below the whiskers indicate values ± 1.5 times the interquartile range. Performance of the two commercial check hybrids is indicated with a *filled circle* and *filled triangle* in *magenta* for CH1 and CH2, respectively. Usefulness for a selection intensity of 10% (U_10 %_) is indicated with *black filled triangles*. Traits are early vigor and early plant height at V4 stage (EV_V4, PH_V4), final plant height (PH_final), female flowering (FF), root lodging (RL), tillering (TILL), dry matter content (DMC), and total dry matter yield (TDMY)

In the following, we will refer to a subset of traits as “core traits” since they are considered most important for improvement of early plant development in elite germplasm. These traits were EV_V4 and PH_V4 as representatives for early development, RL and TILL as representatives for traits for which genetic variation is not acceptable in elite germplasm, PH_final and FF as important agronomic traits, and DMC and TDMY for evaluating yield performance. Phenotypic variation for core traits within and across landraces is shown in Fig. 3 (LP) and Fig. 4 (TC) and for all other traits in Fig. S1 (LP) and Fig. S2 (TC). Phenotypic means, variance components, and heritabilities for all traits are provided in Table S3 and Table S4 for LP and TC performance, respectively. The DH libraries exhibited considerable phenotypic variation for all traits. In LP and TC, a similar range of trait values was observed for all DH libraries. Probably due to the small sample size, distribution of phenotypes in LL deviated slightly from the other two landraces, e.g., for traits EV and TILL. Mean performance differed significantly (*P* < 0.05) across landraces for 20 out of 25 traits in LP and for 5 out of 14 traits in TC, which was a result of the high-quality phenotypic data and large sample sizes of KE and PE. As expected, mean LP performance of the DH libraries was significantly (*P* < 0.05) lower than the respective landraces for almost all traits. The reduction was most pronounced for early development traits, final plant height, and photosynthetic efficiency (Fig. 3, Fig. S1). Flowering time of the DH library was delayed by 10 (LL) and 6 (KE, PE) days compared to the non-inbred material. While the LL DH library had consistently lower mean performance in early development traits, ear height, and final plant height compared to KE and PE, this was not true for the original landraces.

**Fig. 5:**
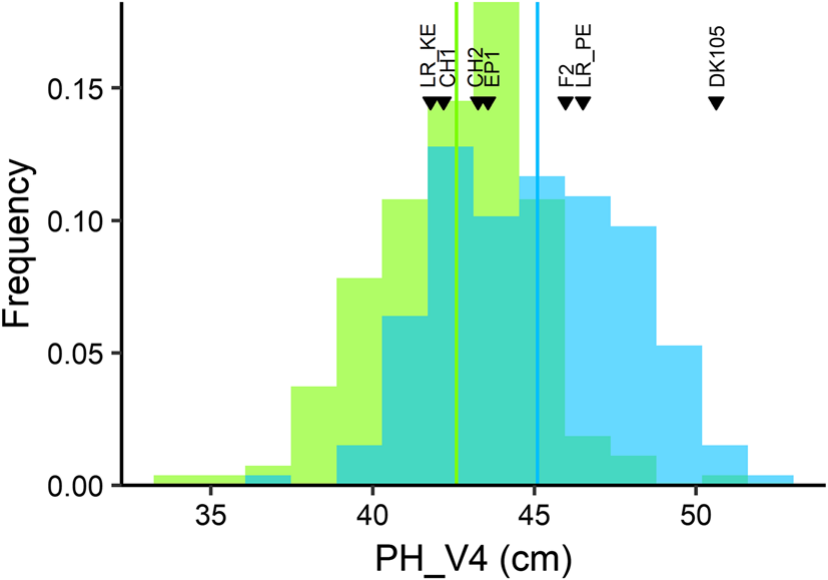
Histogram of testcross (TC) performance of DH lines from landraces KE (*N* = 190, colored in *green*) and PE (*N* = 188, colored in *blue*) for trait early plant height at V4 stage (PH_V4), including the mean of the DH lines per population (*green* and *blue horizontal bar*) and the performance of testcrosses of lines EP1, F2, DK105, the landrace populations (LR_KE, LR_PE), as well as two commercial check hybrids (CH1, CH2) indicated by labeled *black triangles*

When choosing DH lines to be evaluated as TC, we had applied mild selection for flowering time, plant height, and lodging (see “Materials and methods” for details). Mean TC performance of the DH libraries KE and PE did not differ significantly from the TC mean of their respective landrace populations for all traits except for TDMY in PE, indicating that DH lines evaluated as TC represented a random sample of the entire DH library. The TC of many DH lines outperformed the commercial hybrids as well as the TC of founder lines and landraces for the target trait early development, as is shown for PH_V4 in Fig. 5. Only the testcross of inbred DK105 fell into the upper 10% of the distribution of PH_V4. As expected, the commercial hybrids significantly (*P* < 0.05) outperformed the TC mean of DH lines for TDMY by about 15% and, in contrast to the DH lines, showed no TILL or RL (Fig. 4).

Genetic variances were highly significant in LP and TC for most traits under investigation (Table S3, Table S4). Variance component estimates for LL were similar to the other two libraries, but, due to the small sample size, they were estimated with considerably larger error, resulting in nonsignificant genetic variances for PH_V3, TILL, ASI, photosynthesis-related traits, and SPAD. As expected from quantitative genetic theory, genetic variance component estimates were smaller in TC than in LP. In the statistical model, we allowed for heterogeneity of genetic variances estimated within landraces, but only a few traits (e.g., DT, RL) showed strong differences (> twofold) in genetic variance estimates between KE and PE in LP, which were even alleviated in TC.

In LP, trait heritabilities were generally high and similar across landraces, ranging from 0.35 to 0.96. Except for PH_V3, TILL, ASI, Fv/Fm, and SPAD in LL, the heritability estimate always exceeded twice its standard error (Table S3). In TC, heritabilities were slightly lower overall than in LP (Table S4), ranging from 0.31 to 0.92, which was expected from the lower number of testing environments and the lower genetic variance compared to LP.

### Variation across environments

DH libraries were evaluated in a total of 11 environments comprising seven different locations and two years. Locations covered a geographical region spanning from northern Germany to northwestern Spain at altitudes ranging from 29 to 706 m above sea level (Table S1). Average temperatures differed by 5 °C between the coldest (OLI 2017, 14.0 °C) and the warmest (TOM 2018, 19.0 °C) environments, and precipitation varied from 159 (KLW 2018) to 548 mm (ROG 2018) during the vegetation period. The ratio of genotype by environment and genotype variance components depended on the trait under study. In LP, values ranged from 0.11 (EH in KE) to 1.22 (ASI in PE), but varied between 0.2 and 0.7 for most traits with a mean of 0.51 (Table S3). Similar ratios were observed in TC (Table S4).

Correlations between locations for traits measured in at least five environments ranged from 0.40 to 0.87 in 2017 and from 0.19 to 0.86 in 2018 (Table S6). Correlations between years of a given trait and location ranged from 0.31 to 0.83 (Table S6).

### Trait correlations

**Fig. 6:**
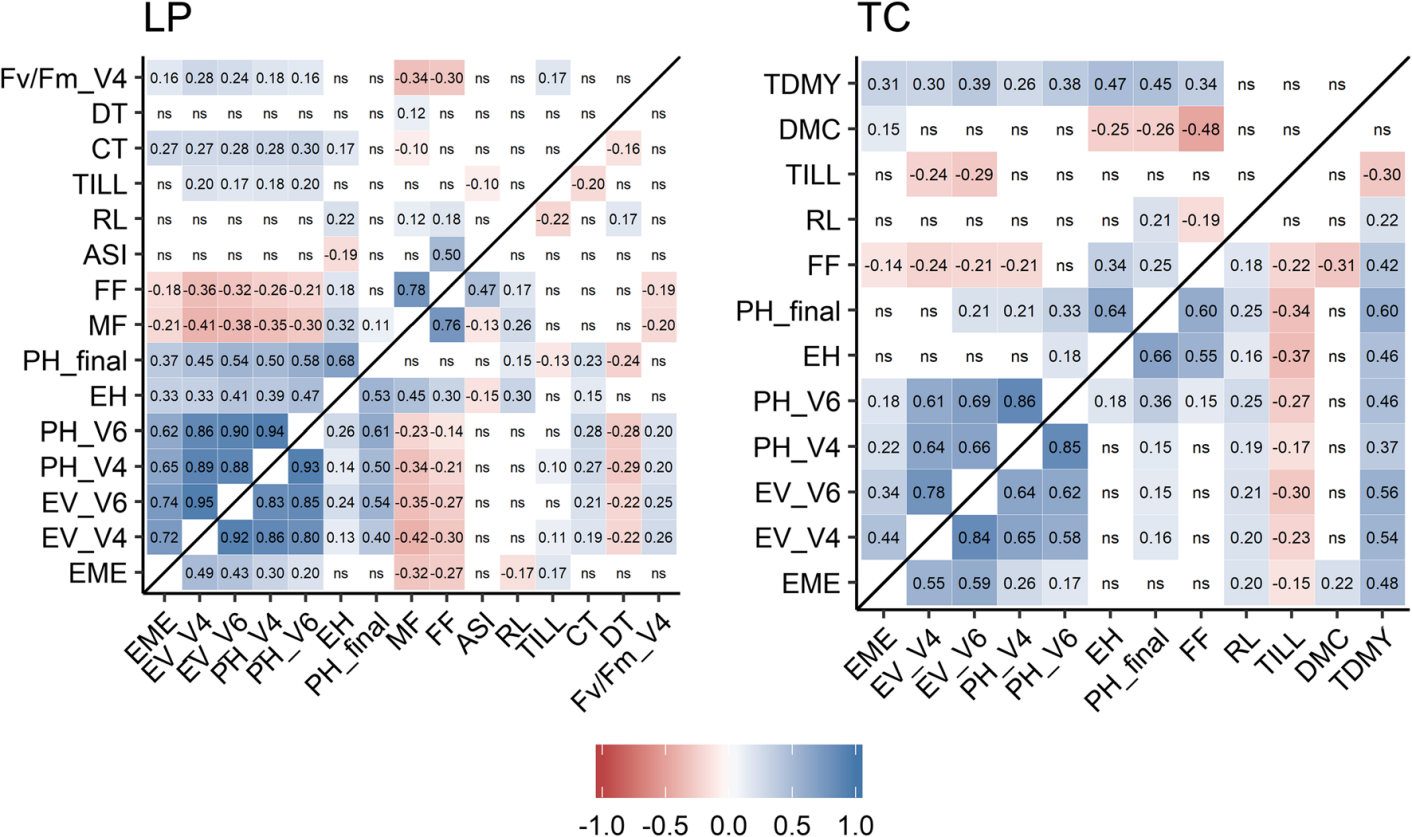
Phenotypic Pearson correlation coefficients for line per se [LP, *left, N* = 471 (KE) and 402 (PE)] and testcross [TC, *right*, *N* = 190 (KE) and 188 (PE)] data within DH libraries KE (above diagonal) and PE (below diagonal) for the traits emergence (EME), early vigor, and early plant height at stages V4 and V6 (EV_V4, EV_V6, PH_V4, PH_V6), ear height (EH), final plant height (PH_final), male flowering and female flowering (MF, FF), anthesis-silking interval (ASI), root lodging (RL), tillering (TILL), cold tolerance (CT), drought/heat tolerance (DT), maximum photosynthetic efficiency at V4 stage (Fv/Fm_V4), dry matter content (DMC), and total dry matter yield (TDMY). *P*-values were adjusted using Bonferroni–Holm correction for multiple testing. Nonsignificant correlations are labeled with ns

In LP and TC, phenotypic correlations among early development traits measured at different growth stages were high and stable across DH libraries, ranging from 0.58 to 0.95 (Fig. 6). The corresponding genetic correlations were slightly higher, ranging from 0.65 to 1 (Fig. S3). For LL in LP, only phenotypic correlations among early development traits (ranging from 0.82 to 0.93, data not shown), PH_final and EH (0.75), and FF and MF (0.69) were significant.

In LP, the early development traits showed intermediate to high positive phenotypic and genetic correlations with final plant height (phenotypic 0.4 to 0.6, genetic 0.4 to 0.7). In TC, only the phenotypic correlation between PH_V6 and final plant height was significant, but it was lower than in LP. Genetic correlations for EV_V4, EV_V6, PH_V4, and PH_V6 with PH_final ranged between 0.2 and 0.5. Intermediate positive correlations were found between early development traits and TDMY in TC (phenotypic 0.3 to 0.6, genetic 0.4 to 0.8) as well as negative correlations of early development with flowering time in LP (FF and MF, phenotypic − 0.2 to − 0.4, genetic − 0.2 to − 0.5) in KE and PE libraries. Phenotypic and genetic correlations of RL with all other traits were nonsignificant or small in LP and TC. The same was true for TILL except for TC of PE, where intermediate correlations with early and late plant height, ear height, and TDMY were observed.

Phenotypic correlations between LP and TC performance were significant for all traits except EME. Genetic correlations between LP and TC were intermediate (early development traits, 0.35 to 0.68) to high (PH_final and FF > 0.78) (Table S5).

## Discussion

Our study is part of a long-term research project which aims to make maize landrace diversity amenable to plant breeding (www.europeanmaize.net). We produced DH libraries from three landraces for obtaining reproducible genetic units for phenotyping and genotyping and characterized them comprehensively to build a publicly available, immortal genetic resource that is ready to use for pre-breeding and for investigations on functional diversity and the prospects of genomic prediction.

### DH libraries capture native diversity for germplasm improvement

The three landraces were chosen to represent the molecular variance of the European landraces characterized by Mayer et al. (2017). Individually, they accounted for more than 75% of the molecular variance in this collection, together for 95%. These findings corroborate results from the literature where it has been shown for several outcrossing species, including maize, that a large proportion of the molecular variation can be found within landraces, while differences between landraces account only for a small proportion (Böhm et al. 2014; Greene et al. 2014; Monteiro et al. 2016). Genotyping with the SNP array technology might have led to an overestimation of the captured molecular variance due to an enrichment of markers with intermediate allele frequencies. For truly quantitative traits, however, the contribution of rare alleles to the additive genetic variance is small and the molecular variance assessed with array data should translate directly into genetic variation observable in phenotypes. With only three (LP) or two (TC) landraces in the statistical model, decomposition of the genetic variance within and across landraces is not meaningful, but from Figs. 3, 4 and Figs. S1, S2 it becomes obvious that differences in trait means across landraces were small compared to the range of values within landraces. Although each landrace accounted for a large proportion of molecular variance individually, we still advise to analyze progenies from several landraces for capturing the genetic variance segregating in a germplasm pool. Molecular variance might be a good indicator for genetic variance averaged across traits, but variation for individual traits must be evaluated for each landrace specifically, as was shown here for TILL, RL, DT, and CT. Different landraces may also differ with respect to their success rates in DH production (Melchinger et al. 2017), pointing to different multiplication histories. While KE and PE may have encountered bottlenecks or inbreeding in the past, LL seems to carry a much higher genetic load that limited the production of fully homozygous DH lines for this landrace. This assumption is also supported by the significantly lower LP mean performance of the LL DH library for early development, ear height, and final plant height compared to KE and PE that was not observed for the original landraces.

The DH libraries generated in this study represented their respective landraces accurately in terms of molecular variance. DH lines and S_0_ gametes from the same landrace overlapped nicely in the PCoA (Fig. 1) and the AMOVA showed that almost all molecular variation was found within S_0_ gametes and DH lines (> 95%) and not between them. Individuals sampled from a maize landrace are assumed to be unrelated, but pairwise comparisons share different numbers of alleles alike in state, leading to variation in similarity between them. Patterns of variation in similarity were comparable for S_0_ plants and DH lines (Fig. 2), corroborating that the two types of progeny represent their original landraces in a similar way. We thus conclude that the three DH libraries derived from KE, PE, and LL represent a valuable resource for genetic improvement of elite flint germplasm, since they cover a large proportion of the genomic and genetic variance of the landrace collection described in Mayer et al. (2017).

### Improving early plant development

In many growing regions worldwide, maize encounters low to moderate temperatures during the early vegetative phase. Under these conditions, accelerated early development can increase final biomass yield. Genetic enhancement of early growth can also improve resource efficiency, preserve soil fertility, and reduce the need for herbicide treatment. European flint maize germplasm has been adapted to the temperate climate conditions of Northern and Central Europe through breeding, but genetic variation for early development under cool temperatures has been depleted simultaneously (Greaves 1996; Rodríguez et al. 2010).

In LP and TC of the DH libraries, the target trait early development assessed through early vigor scores and early plant height measurements showed ample genetic variation (Figs. 3, 4, Figs. S1, S2). In TC, the majority of DH lines outperformed the commercial hybrids for PH_V4, and only one check (F353 × DK105) ranged among the best 10% DH lines, suggesting that the DH libraries can serve as a valuable source of alleles for improving early development traits of the elite European flint pool (Fig. 5).

Identifying maize flint germplasm with superior early growth has been the objective of several studies in both, field and controlled environments (Peter et al. 2009a, b; Rodríguez et al. 2010; Revilla et al. 2016). In most studies, early development was assessed as a visual score, which delivers ordinal endpoints and can be rather subjective. On the other hand, early plant height measurements consume considerable resources. Early vigor scores showed a substantially higher correlation with plant emergence compared to early plant height in this research (Fig. 6, Fig. S3). Even though all TC seed was produced on inbred line F353, the higher phenotypic and genetic correlation of early vigor and EME was maintained. For PH_V4, the commercial hybrids were on average not different from the TC mean of the DH libraries, but they scored better for EV. Thus, the early plant height measurement neglects information that can be accounted for by EV scores, such as differences in leaf coloration or the overall lower EME of the DH library testcrosses. In addition, genetic correlations between TDMY and EV were substantially higher compared to between TDMY and PH_V4 supporting the hypothesis that, although highly correlated, the two types of measurements target different components of early development. For a comprehensive characterization of early growth development, it seems advisable to assess both, EV and early plant height. To allow dissection of early growth development into its genetic components and consequently provide a better understanding of the underlying genetic mechanisms, we propose establishing growth models by monitoring early development at high resolution in time using remote sensing in the field (Huang et al. 2013; Bendig et al. 2015) or in controlled conditions (Gioia et al. 2017). The three DH libraries KE, PE, and LL are most suitable for further investigation on this topic as they exhibit more pronounced genetic variation in early growth traits than can be expected from elite material (Revilla et al. 1999; Peter et al. 2009a).

### Comprehensive phenotypic characterization of DH libraries

The prospects for the genetic improvement of elite germplasm for early growth development through the use of landrace-derived material have to be evaluated in a multi-trait context. Comprehensive data on trait correlations are crucial in order to avoid undesired selection response in traits of agronomic importance.

In LP, EV_V4 and PH_V4 showed intermediate negative genetic correlations with flowering time and positive genetic correlations with PH_final, corroborating results of Böhm et al. (2017) on DH lines derived from landraces. Thus, selection for accelerated early development will lead to increased plant height and early flowering which, depending on the target environment, might not be desirable. The DH libraries also showed variation for RL and TILL. Given the low levels of genetic correlations with early development traits and the usefulness of the best 10% of DH lines being close to zero, a simultaneous reduction or removal of lodging and tillering should be possible in a recurrent selection program devoted to the improvement of early development traits. In TC, correlations between early development traits and TDMY were positive. However, the commercial hybrids significantly outperformed the DH lines for TDMY, while testcrosses of founder lines (F2, EP1, DK105) lay well within the range of the DH libraries for both traits (Fig. 4, Fig. S4). The yield gap between the mean testcross yield of the DH lines and the mean testcross yield of two commercial hybrids amounted to about 15% and was comparable to what was reported in the literature for other European landraces (Wilde et al. 2010; Brauner et al. 2019). The usefulness of the best 10% DH lines in KE and PE, respectively, remained 8% below the performance level of the commercial hybrids for TDMY (Fig. 4). Given that the inbred line F353 used as tester for the DH libraries was developed about 20 years ago (year of release 2001, C. Bauland, personal communication) and that the parental components of the commercial check hybrids were highly selected based on their general and specific combining ability, the difference in TDMY between commercial hybrids and the top 10% DH lines seems small and could likely be reduced by the use of modern testers (Hölker et al. 2019). In many material groups, a negative correlation between DMC and TDMY is expected. In our research, phenotypic correlations between TDMY and DMC were nonsignificant when averaged across environments (Fig. 6, Fig. S4) as well as in all four individual environments where TC performance was evaluated (data not shown). This outcome can most likely be attributed to the exceptionally hot and dry conditions during the 2018 growing season (Table S1), the genetic material under study, or an interaction of both. Thus, an additional year of TC evaluation, including more and also later maturing commercial check hybrids, will be conducted for investigating the DMC/TDMY relationship in material derived from genetic resources more closely and for evaluating the overall yield potential of the DH libraries.

### Multi-environment testing

One of the aims of this study was to assess trait differentiation in diverse environments and to estimate the magnitude of genotype × environment interactions of landrace-derived material. Thus, the chosen environments covered a broad spectrum of target regions for European flint material (Table S1). Despite locations with very different climatic conditions (e.g., OLI and TOM) and large differences in temperature and precipitation in 2017 and 2018, the ratio of genotype × environment and genetic variance ($\sigma_{gu}^{2} :\; \sigma_{g}^{2}$, Tables S3, S4) was moderate for most traits. If landraces from which DH libraries are derived are adapted to similar environmental conditions as the target elite breeding germplasm, the confounding effects of adaptive alleles and strong genotype × environment interactions can be avoided and meaningful phenotypes obtained. Thus, our results are encouraging with respect to the prospects of incorporating environmentally stable alleles from pre-selected DH libraries into elite germplasm.

Evaluating landrace-derived material in 11 environments might not be practicable for applied pre-breeding programs. In this study, the large number of test environments was highly useful because we detected the segregation of unfavorable alleles in specific environments such as segregation for rust in TOM (Fig. S5) and drought susceptibility in EIN (Fig. S6), both in 2018. Although infections with rust or severe drought may not occur frequently, it would be devastating if these susceptibilities were transferred inadvertently to elite germplasm through the introgression of landrace-derived material. If evaluating the landrace-derived material in a large number of environments is not possible, prioritized testing in environments known for high disease pressure, abiotic stress, or frequent occurrence of undesirable traits like RL is highly advisable.

### DH libraries from landraces make native diversity accessible

The DH libraries presented in this study link the large molecular diversity present in landraces to meaningful phenotypes. DH lines from landraces outperformed flint founder lines and commercial hybrids in early development, and as immortal genetic units they are directly accessible for plant breeding. Improving one or several target traits and simultaneously closing the performance gap between elite and landrace-derived genetic material for multiple traits of agronomic importance requires efficient recurrent population improvement. In this context, knowledge of trait correlations is crucial in order to broaden the narrow genetic base of the elite flint germplasm pool without introducing undesired traits from landraces into elite breeding populations. To obtain maximum selection gain per unit time, theory offers different strategies, such as multi-stage or index selection (Bernardo 2002), which need to be evaluated in the framework of the respective breeding programs. Optimal strategies may vary conditional on species, budget, and short-term or long-term perspectives. Böhm et al. (2017) suggested multi-stage phenotypic selection of landrace-derived DH libraries. In a simulation study, Gorjanc et al. (2016) compared different scenarios for initiating pre-breeding for maize landraces using genomic prediction (GP) and suggested starting directly from landraces (e.g., without crossing to elite lines).

The implementation of GP in pre-breeding of landrace-derived material is still underexploited. The comprehensive phenotypic data and derived quantitative genetic parameters presented for the three DH libraries in this study provide an excellent basis for optimizing genome-based pre-breeding schemes. Multi-environment phenotypic data are available for model training in LP and TC. Sample sizes and marker densities are large, allowing to investigate the effects of population size and required marker densities in populations with relatively low linkage disequilibrium compared to elite germplasm. In addition to investigating the prospects of genome-based prediction, our data provide a comprehensive framework for the discovery of genes controlling favorable and unfavorable traits as well as for the genetic analysis of additional relevant traits such as nutrient efficiency, photosynthesis-related traits, and additional biotic and abiotic stress tolerances.

## Electronic supplementary material

Below is the link to the electronic supplementary material.

Supplementary material 1 (PDF 1613 kb)
