# Supplementary material for: European maize landraces made accessible for plant breeding and genome-based studies
Source: Theor Appl Genet. 2019 Sep 26;132(12):3333–45. doi: 10.1007/s00122-019-03428-8 (PMC6820615; doi:10.1007/s00122-019-03428-8)
Supplement: Supplementary file 1 — Supplementary material 1 (PDF 1613 kb) [file 122_2019_3428_MOESM1_ESM.pdf]

Theoretical and Applied Genetics

European maize landraces made accessible for plant breeding and genome-based studies

Armin C. Hölker[1], Manfred Mayer[1], Thomas Presterl[2], Therese Bolduan[2], Eva Bauer[1], Bernardo Ordas[3], Pedro C. Brauner[4,†], Milena Ouzunova[2], Albrecht E. Melchinger[4], Chris-Carolin Schön[*1]

[1] Plant Breeding, TUM School of Life Sciences Weihenstephan, Technical University of Munich, Freising, Germany, 85354

[2] Maize Breeding, KWS Saat SE, Einbeck, Germany, 37574

[3] Misión Biológica de Galicia, Spanish National Research Council (CSIC), Pontevedra, Spain, 36080

[4] Institute of Plant Breeding, Seed Science and Population Genetics, University of Hohenheim, Stuttgart, Germany, 70593

[†] present address: Maize Breeding, KWS Saat SE, Einbeck, Germany, 37574

[*]Corresponding author

Email address: chris.schoen@tum.de

Supplemental tables

Table S1 Geographic coordinates and altitude (meters above sea level, m a.s.l.) for the field trial locations Einbeck (EIN, Germany), Roggenstein (ROG, Germany), Bernburg (BBG, Germany), Klein Wanzleben (KLW, Germany), Oberer Lindenhof (OLI, Germany), Tomeza (TOM, Spain), and Golada (GOL, Spain). Weather data were obtained for the period from 15th of April to 30th of September in each year from the mentioned nearby weather station. The approximate distance to field trial locations is given. Weather data contains total precipitation and average, minimum and maximum daily temperature in the aforementioned period of each year.

Location	Coordinates	Altitude (m a.s.l.)	Location weather station	Distance (km)	Year	Precipitation (mm)	Average temperature (°C)	Minimum temperature (°C)	Maximum temperature (°C)
EIN	51.81831, 9.86674	116	Lutterbeck[a]	11	2017	495	14.6	-3.6	32.7
					2018	188	17.1	0.9	35.5
ROG	48.17985, 11.32025	537	Roggenstein[b]	0	2017	543	15.3	-2.9	33.5
					2018	548	17.2	-0.8	33.8
BBG	51.8246, 11.70731	84	Bernburg[a]	0	2017	304	16.7	-3.0	33.3
					2018	174	18.9	0.7	39.5
KLW	52.06887, 11.36615	117	Magdeburg[a]	15	2017	268	16.3	-3.3	32.1
					2018	159	18.6	2.0	37.3
OLI	48.47396, 9.30498	706	Oberer Lindenhof[c]	0	2017	524	14.0	-6.0	34.6
					2018	314	15.7	-0.3	36.2
TOM	42.41072, -8.63441	29	Pontevedra[a]	4	2017	279	18.5	3.5	35.9
					2018	241	19.0	3.3	37.7
GOL	42.77536, -8.10608	335	Santiago de Compostela[a]	28	2017	234	17.5	1.6	36.2
					2018	168	17.6	1.3	36.2

[a] Data obtained from the Global Historical Climatology Network (Menne et al. 2012).
[b] Data obtained from Agrarmeteorologie Bayern, Bavarian State Research Center for Agriculture, station Roggenstein.
[c] Data obtained from Agrarmeteorologie Baden-Württemberg, Landwirtschaftliches Technologiezentrum Augustenberg, station Oberer Lindenhof.

Table S2 Table specifying which traits have been measured in which environments for line per se (LP) and testcross (TC) trials. Traits are emergence (EME), early vigor and early plant height at stages V3, V4, and V6 (EV_V3, EV_V4, EV_V6, PH_V3, PH_V4, PH_V6), ear height (EH), final plant height (PH_final), male flowering (MF), female flowering (FF), anthesis-silking-interval (ASI), root lodging (RL), tillering (TILL), cold tolerance (CT), drought/heat tolerance (DT), tassel length (TL), spike length (SL), number of tassel branches (NB), tassel angle (TA), maximum photosystem II efficiency at stages V4 and V6 (Fv/Fm_V4, Fv/Fm_V6), leaf greenness at stages V3, V4, and V6 (SPAD_V3, SPAD_V4, SPAD_V6), dry matter content (DMC), and total dry matter yield (TDMY).

Trait	Environments LP	Environments TC
EME	All 11	All 4
EV_V3	All except EIN 2018	KLW, OLI, ROG
EV_V4	All 11	All 4
EV_V6	All 11	All 4
PH_V3	GOL and TOM 2017 and 2018	KLW and OLI
PH_V4	All 11	All 4
PH_V6	All 11	All 4
EH	ROG 2017 and 2018	ROG
PH_final	All 11	All 4
MF	EIN and TOM 2017 and 2018, GOL 2018	
FF	All except GOL 2017	EIN, OLI, ROG
ASI	EIN and TOM 2017 and 2018, GOL 2018	
RL	BBG 2017, EIN 2017 and 2018, OLI 2017, ROG 2017 and 2018	EIN, OLI, ROG
TILL	EIN and ROG 2017 and 2018, KLW 2018	OLI
CT	OLI 2017	
DT	EIN 2018	
TL	ROG 2018	
SL	ROG 2018	
NB	ROG 2018	
TA	ROG 2018	
Fv/Fm_V4	GOL and TOM; 2017 and 2018	
Fv/Fm_V6	GOL and TOM 2017	
SPAD_V3	GOL and TOM; 2017 and 2018	
SPAD_V4	GOL and TOM; 2017 and 2018	
SPAD_V6	GOL and TOM 2017	
DMC		All 4
TDMY		All 4

Table S3 Phenotypic means ($\bar{X}$), genotype (σ_g^2) and genotype × environment (σ_{gu}^2) variance components and their ratio ($\sigma_{gu}^2 : \sigma_g^2$), and heritability ($h^2$) including standard errors for line per se (LP) evaluation in landraces KE, PE, and LL. Traits are emergence (EME), early vigor and early plant height at stages V3, V4, and V6 (EV_V3, EV_V4, EV_V6, PH_V3, PH_V4, PH_V6), ear height (EH), final plant height (PH_final), male flowering (MF), female flowering (FF), anthesis-silking-interval (ASI), root lodging (RL), tillering (TILL), cold tolerance (CT), drought/heat tolerance (DT), tassel length (TL), spike length (SL), number of tassel branches (NB), tassel angle (TA), maximum photosystem II efficiency at V4 and V6 stage (Fv/Fm_V4, Fv/Fm_V6), and leaf greenness at stages V3, V4 and V6 (SPAD_V3, SPAD_V4, SPAD_V6). The number of environments (#E) in which the trait was measured is given.

		LP												
Trait	LR	$\bar{X}$[a]	±	SE	σ_g^2	±	SE	σ_{gu}^2	±	SE	$\sigma_{gu}^2 : \sigma_g^2$	h^2	±	SE
EME (%) (#E = 11)	KE	69[A]	±	2	257	±	18	62	±	2.8	0.24	0.96	±	0.003
	PE	76[B]	±	2	167	±	13	51	±	2.6	0.31	0.90	±	0.013
	LL	70[AB]	±	4	212	±	71	152	±	25	0.72	0.96	±	0.013
EV_V3 (#E = 10)	KE	4.9[A]	±	0.2	0.83	±	0.06	0.25	±	0.01	0.30	0.95	±	0.004
	PE	5.4[B]	±	0.2	0.48	±	0.04	0.23	±	0.01	0.48	0.92	±	0.013
	LL	4.6[A]	±	0.3	0.87	±	0.28	0.20	±	0.06	0.23	0.95	±	0.015
EV_V4 (#E = 11)	KE	5.0[A]	±	0.2	0.78	±	0.06	0.30	±	0.01	0.38	0.94	±	0.004
	PE	5.3[B]	±	0.2	0.45	±	0.04	0.33	±	0.02	0.73	0.88	±	0.017
	LL	4.7[A]	±	0.3	0.68	±	0.24	0.48	±	0.10	0.71	0.93	±	0.022
EV_V6 (#E = 11)	KE	5.3[A]	±	0.3	0.66	±	0.05	0.33	±	0.01	0.50	0.93	±	0.005
	PE	5.5[A]	±	0.3	0.42	±	0.04	0.34	±	0.01	0.81	0.86	±	0.019
	LL	4.7[B]	±	0.3	0.69	±	0.25	0.58	±	0.10	0.84	0.93	±	0.023
PH_V3 (cm) (#E = 4)	KE	22[A]	±	3	11.7	±	1.2	2.52	±	0.3	0.22	0.91	±	0.010
	PE	25[BC]	±	3	8.0	±	0.9	2.14	±	0.3	0.27	0.94	±	0.001
	LL	22[AC]	±	4		ns			ns				ns	
PH_V4 (cm) (#E = 11)	KE	40[A]	±	6	30.0	±	2.2	11.4	±	0.5	0.38	0.95	±	0.004
	PE	44[B]	±	6	24.7	±	2.0	18.4	±	0.7	0.74	0.91	±	0.012
	LL	38[A]	±	6	15.9	±	6.1	18.4	±	3.5	1.16	0.87	±	0.043
PH_V6 (cm) (#E = 11)	KE	78[A]	±	8	90	±	6	28	±	1	0.31	0.95	±	0.004
	PE	83[B]	±	8	77	±	6	39	±	2	0.51	0.90	±	0.014
	LL	72[C]	±	8	63	±	23	72	±	12	1.14	0.92	±	0.028
EH (cm) (#E = 2)	KE	62[A]	±	7	185	±	14	20	±	3	0.11	0.90	±	0.011
	PE	49[B]	±	7	129	±	11	22	±	4	0.17	0.87	±	0.049
	LL	42[B]	±	8	203	±	68		ns			0.90	±	0.030
PH_final (cm) (#E = 11)	KE	141[A]	±	7	327	±	23	101	±	4	0.31	0.96	±	0.003
	PE	125[B]	±	7	284	±	21	91	±	4	0.32	0.96	±	0.006
	LL	116[C]	±	7	207	±	67	85	±	19	0.41	0.94	±	0.018
FF (d) (#E = 10)	KE	80[ns]	±	2	16.4	±	1.2	4.3	±	0.2	0.26	0.96	±	0.003
	PE	79[ns]	±	2	14.8	±	1.1	4.2	±	0.2	0.28	0.93	±	0.010
	LL	78[ns]	±	2	14.2	±	4.7	7.6	±	1.5	0.54	0.95	±	0.015
MF (d) (#E = 5)	KE	77[A]	±	2	13.9	±	1.1	3.0	±	0.2	0.22	0.93	±	0.006
	PE	75[B]	±	2	12.6	±	1.1	4.0	±	0.3	0.32	0.86	±	0.034
	LL	75[B]	±	2	12.4	±	5.4	8.3	±	2.6	0.67	0.91	±	0.036
ASI	KE	4[ns]	±	2	4.3	±	0.5	4.7	±	0.4	1.09	0.72	±	0.029

(d) (#E = 5)	PE	5^{ns} ± 2	4.9 ± 0.6	6.0 ± 0.5	1.22	0.67 ± 0.065
	LL	5^{ns} ± 2	ns	8.1 ± 3.2		ns
RL (#E = 6)	KE	2.8^{A} ± 0.4	2.0 ± 0.2	1.2 ± 0.06	0.60	0.86 ± 0.011
	PE	1.8^{B} ± 0.4	0.6 ± 0.1	0.5 ± 0.05	0.83	0.58 ± 0.047
	LL	3.2^{A} ± 0.5	1.8 ± 0.7	1.7 ± 0.41	0.94	0.89 ± 0.037
TILL (#E = 5)	KE	2.4^{A} ± 0.3	1.1 ± 0.08	0.3 ± 0.02	0.27	0.90 ± 0.008
	PE	3.7^{B} ± 0.3	2.0 ± 0.15	0.4 ± 0.03	0.20	0.94 ± 0.011
	LL	1.4^{C} ± 0.3	ns	0.4 ± 0.11		ns
CT (#E = 1)	KE	3.9^{ns} ± 0.2	1.8 ± 0.17			0.73 ± 0.024
	PE	4.0^{ns} ± 0.2	1.4 ± 0.16			0.69 ± 0.028
	LL	3.9^{ns} ± 0.6	4.0 ± 1.95			0.86 ± 0.059
DT (#E = 1)	KE	8.9^{A} ± 0.04	0.1 ± 0.02			0.46 ± 0.049
	PE	7.9^{B} ± 0.09	2.4 ± 0.18			0.95 ± 0.004
	LL	8.1^{B} ± 0.32	2.4 ± 0.72			0.96 ± 0.013
TL (cm) (#E = 1)	KE	30.9^{A} ± 0.22	14.4 ± 1.3			0.83 ± 0.016
	PE	36.2^{B} ± 0.26	20.2 ± 1.7			0.88 ± 0.012
	LL	28.8^{C} ± 0.89	17.1 ± 5.8			0.86 ± 0.042
SL (cm) (#E = 1)	KE	22.5^{A} ± 0.23	12.0 ± 1.1			0.83 ± 0.016
	PE	23.7^{B} ± 0.24	14.0 ± 1.2			0.85 ± 0.014
	LL	20.5^{C} ± 0.77	12.3 ± 4.3			0.83 ± 0.051
NB (#E = 1)	KE	11^{A} ± 0.25	19.7 ± 1.7			0.89 ± 0.011
	PE	16^{B} ± 0.25	19.2 ± 1.6			0.88 ± 0.011
	LL	9^{A} ± 0.84	15.7 ± 5.2			0.86 ± 0.040
TA (#E = 1)	KE	3.6^{A} ± 0.07	0.93 ± 0.09			0.80 ± 0.019
	PE	4.2^{B} ± 0.06	0.89 ± 0.09			0.79 ± 0.019
	LL	3.6^{A} ± 0.18	0.53 ± 0.22			0.69 ± 0.090
Fv/Fm_V4[b] (#E = 4)	KE	0.72^{ns} ± 0.01	0.4 ± 0.06	0.2 ± 0.04	0.50	0.76 ± 0.028
	PE	0.74^{ns} ± 0.01	0.3 ± 0.05	0.2 ± 0.04	0.67	0.58 ± 0.085
	LL	0.73^{ns} ± 0.01	ns	ns		ns
Fv/Fm_V6[b] (#E = 2)	KE	0.71^{ns} ± 0.01	0.6 ± 0.10	ns		ns
	PE	0.73^{ns} ± 0.01	0.3 ± 0.08	0.2 ± 0.09	0.67	0.35 ± 0.147
	LL	0.73^{ns} ± 0.01	ns	ns		ns
SPAD_V3 (#E = 4)	KE	11.9^{A} ± 1.5	5.1 ± 0.61	1.7 ± 0.28	0.33	0.82 ± 0.020
	PE	13.1^{BC} ± 1.5	5.6 ± 0.67	2.8 ± 0.22	0.50	0.83 ± 0.055
	LL	12.8^{AC} ± 1.9	ns	ns		ns
SPAD_V4 (#E = 4)	KE	12.0^{A} ± 1.8	7.2 ± 0.85	2.0 ± 0.39	0.28	0.83 ± 0.020
	PE	14.2^{B} ± 1.8	7.0 ± 0.84	3.3 ± 0.45	0.47	0.53 ± 0.088
	LL	15.6^{B} ± 2.0	ns	20 ± 8.12		ns
SPAD_V6 (#E = 2)	KE	19.6^{A} ± 0.7	22 ± 3.1	6.5 ± 1.8	0.30	0.73 ± 0.040
	PE	21.5^{B} ± 0.7	17 ± 2.8	8.0 ± 2.0	0.47	0.77 ± 0.031
	LL	23.7^{B} ± 1.8	ns	ns		ns

[a] Means with a common letter are not significantly different (P < 0.05).
[b] Variance components multiplied by 1000.

Table S4 Phenotypic means ($\bar{X}$), genotype (σ_g^2) and genotype × environment (σ_{gu}^2) variance components and their ratio ($\sigma_{gu}^2 : \sigma_g^2$), and heritability ($h^2$) including standard errors for testcross (TC) evaluation of KE and PE. Traits are emergence (EME), early vigor and early plant height at stages V3, V4, and V6 stages (EV_V3, EV_V4, EV_V6, PH_V3, PH_V4, PH_V6), ear height (EH), final plant height (PH_final), female flowering (FF), root lodging (RL), tillering (TILL), dry matter content (DMC) and total dry matter yield (TDMY). The number of environments (#E) in which the trait was measured is given.

		TC												
Trait	LR	$\bar{X}^{a}$	±	SE	σ_g^2	±	SE	σ_{gu}^2	±	SE	$\sigma_{gu}^2 : \sigma_g^2$	h^2	±	SE
EME (%, #E = 4)	KE	87^{A}	±	2.7	10	±	1.3		ns			0.80	±	0.022
	PE	83^{B}	±	2.7	35	±	4.0	2.2	±	0.8	0.06	0.92	±	0.010
EV_V3 (#E = 3)	KE	5.6^{ns}	±	0.3	0.15	±	0.02	0.05	±	0.02	0.33	0.72	±	0.038
	PE	6.1^{ns}	±	0.3	0.10	±	0.02	0.03	±	0.02	0.30	0.65	±	0.047
EV_V4 (#E = 4)	KE	5.7^{ns}	±	0.3	0.14	±	0.02	0.08	±	0.02	0.57	0.68	±	0.041
	PE	4.0^{ns}	±	03.	0.18	±	0.03	0.07	±	0.02	0.39	0.73	±	0.034
EV_V6 (#E = 4)	KE	5.6^{ns}	±	0.4	0.13	±	0.02	0.10	±	0.02	0.77	0.63	±	0.046
	PE	6.0^{ns}	±	0.4	0.17	±	0.03	0.06	±	0.02	0.35	0.71	±	0.036
PH_V3 (cm, #E = 2)	KE	29^{ns}	±	6	2.67	±	0.43		ns			0.69	±	0.048
	PE	31^{ns}	±	6	1.90	±	0.39	0.89	±	0.33	0.47	0.58	±	0.066
PH_V4 (cm, #E = 4)	KE	43^{A}	±	3	4.45	±	0.63		ns			0.76	±	0.030
	PE	45^{B}	±	3	4.95	±	0.70		ns			0.77	±	0.029
PH_V6 (cm, #E = 4)	KE	84^{A}	±	5	11.3	±	1.6	2.6	±	1.0	0.23	0.77	±	0.029
	PE	89^{B}	±	5	13.5	±	1.8		ns			0.81	±	0.024
EH (cm, #E = 1)	KE	155^{ns}	±	4	106	±	15					0.75	±	0.034
	PE	154^{ns}	±	4	98	±	15					0.73	±	0.036
PH_final (cm, #E = 4)	KE	246^{ns}	±	17	86	±	10	15	±	3.5	0.17	0.87	±	0.016
	PE	244^{ns}	±	17	102	±	12	13	±	3.4	0.13	0.90	±	0.013
FF (d, #E = 3)	KE	79^{ns}	±	1	1.6	±	0.2	0.5	±	0.1	0.31	0.82	±	0.024
	PE	79^{ns}	±	1	2.6	±	0.3	0.6	±	0.1	0.23	0.87	±	0.017
RL (#E = 3)	KE	1.8^{ns}	±	0.2	0.28	±	0.05	0.18	±	0.04	0.64	0.63	±	0.048
	PE	2.0^{ns}	±	0.2	0.16	±	0.04	0.38	±	0.05	2.38	0.41	±	0.077
TILL (#E = 1)	KE	2.0^{A}	±	0.1	0.3	±	0.1					0.31	±	0.092
	PE	4.1^{B}	±	0.2	2.4	±	0.3					0.79	±	0.028
DMC (%, #E = 4)	KE	35^{A}	±	1.6	2.2	±	0.3	0.6	±	0.1	0.27	0.83	±	0.021
	PE	34^{B}	±	1.6	2.3	±	0.3	0.9	±	0.1	0.39	0.81	±	0.023
TDMY (dt/ha, #E = 4)	KE	193^{ns}	±	12	89	±	13	37	±	10	0.42	0.72	±	0.035
	PE	192^{ns}	±	12	120	±	17	46	±	10	0.38	0.76	±	0.029

[a] Means with a common letter are not significantly different ($P < 0.05$).

Table S5 Phenotypic Spearman's correlation coefficients (r_p) and genetic correlation (r_g) between line per se (LP) and testcross (TC) performance of landraces KE and PE for traits emergence (EME), early vigor at stages V3, V4, and V6 (EV_V3, EV_V4, EV_V6), early plant height at stages V3, V4 and V6 (PH_V4, PH_V4, PH_V6), final plant height (PH_final), female flowering (FF), root lodging (RL), and tillering (TILL, only phenotypic correlation).

	KE		PE	
	r_p	r_g	r_p	r_g
EME	ns	ns	ns	ns
EV_V3	0.27	0.46	0.25	0.42
EV_V4	0.35	0.54	0.22	0.37
EV_V6	0.41	0.66	0.25	0.35
PH_V3	0.38	0.55	0.40	0.59
PH_V4	0.47	0.62	0.39	0.59
PH_V6	0.48	0.68	0.52	0.65
PH_final	0.68	0.78	0.59	0.80
FF	0.63	0.81	0.61	0.80
RL	0.40	0.89	0.29	0.67
TILL	0.42		0.62	

Table S6 Range (minimum and maximum) of Pearson correlation coefficients for line per se (LP) performance between locations within the years 2017 and 2018 as well as between same locations across years. Traits are emergence (EME), early vigor and early plant height at stages V3, V4, and V6 (EV_V3, EV_V4, EV_V6, PH_V3, PH_V4, PH_V6), ear height (EH), final plant height (PH_final), male flowering (MF), female flowering (FF), anthesis-silking interval (ASI), root lodging (RL), and tillering (TILL).

	Within 2017	Within 2018	Across years
EME	0.75 – 0.85	0.37 – 0.86	0.42 – 0.82
EV_V3	0.61 – 0.78	0.43 – 0.61	0.46 – 0.63
EV_V4	0.51 – 0.77	0.32 – 0.64	0.45 – 0.71
EV_V6	0.40 – 0.71	0.28 – 0.62	0.31 – 0.73
PH_V4	0.59 – 0.82	0.49 – 0.71	0.61 – 0.78
PH_V6	0.58 – 0.84	0.54 – 0.75	0.56 – 0.81
PH_final	0.52 – 0.87	0.49 – 0.79	0.72 – 0.80
MF	0.72 – 0.72	0.54 – 0.75	0.73 – 0.73
FF	0.51 – 0.81	0.37 – 0.71	0.63 – 0.76
ASI	0.47 – 0.47	0.19 – 0.38	0.40 – 0.57
RL	0.55 – 0.62	0.34 – 0.34	0.40 – 0.40
TILL	0.77 – 0.77	0.67 – 0.78	0.77 – 0.83

Supplemental figures

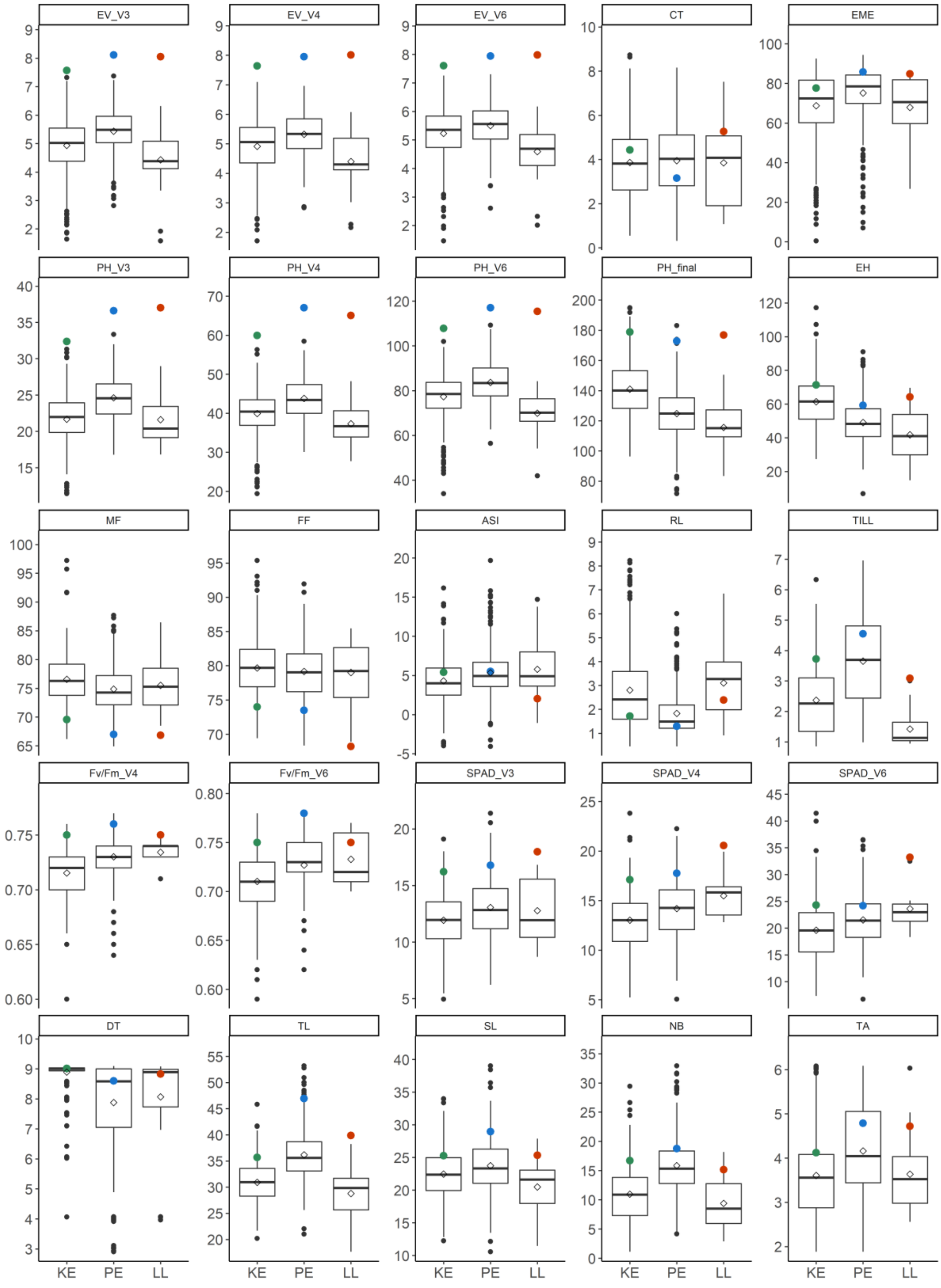

Fig. S1 Boxplots of phenotypic data for line per se (LP) performance for the DH libraries from landraces KE, PE, and LL. Boxplots show the upper and lower quartile, median (*horizontal bar*), mean (*open diamond*), whiskers (*vertical bars*) and the performance of the respective landrace (*filled circle* in *green*, *blue* and *red* for KE, PE and LL, respectively). Points above and below the whiskers indicate values ± 1.5 times the interquartile range. Traits are early vigor at stages V3, V4, and V6 (EV_V3, EV_V4, EV_V6), cold tolerance (CT), emergence (EME), early plant height at stages V3, V4, and V6 (PH_V3, PH_V4, PH_V6), final plant height (PH_final), ear height (EH), male flowering (MF), female flowering (FF), anthesis-silking-interval (ASI), root lodging (RL), tillering (TILL), maximum photosystem II efficiency at V4 and V6 stages (Fv/Fm_V4, Fv/Fm_V6), leaf greenness at stages V3, V4 and V6 (SPAD_V3, SPAD_V4, SPAD_V6), drought/heat tolerance (DT), tassel length (TL), spike length (SL), number of tassel branches (NB), and tassel angle (TA).

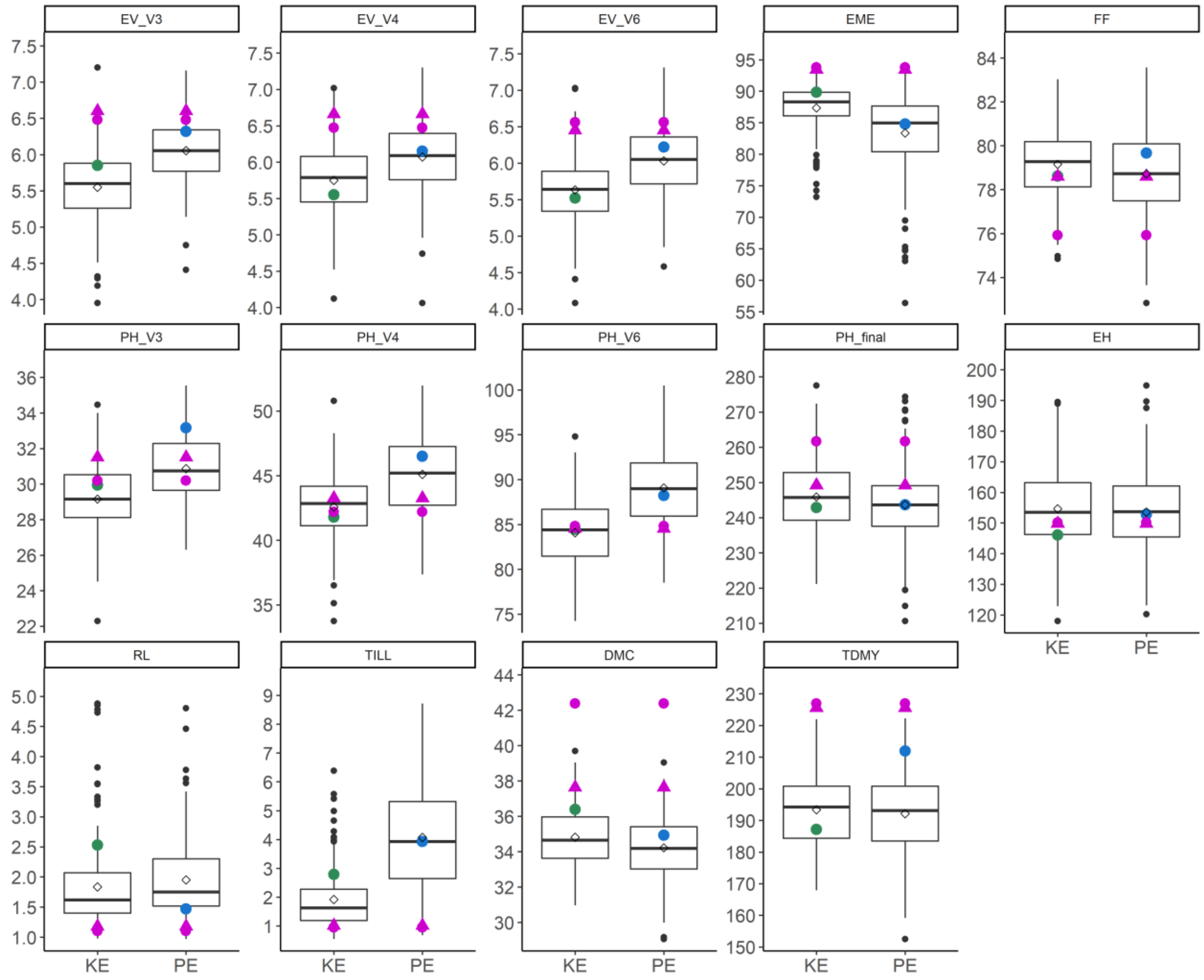

Fig. S2 Boxplots of phenotypic data for testcross (TC) performance for DH libraries from landraces KE and PE. Boxplots show the upper and lower quartile, median (*horizontal bar*), mean (*open diamond*), whiskers (*vertical bars*) and the performance of the respective landrace (*filled circle* in *green* and *blue* for KE and PE, respectively). Points above and below the whiskers indicate values ± 1.5 times the interquartile range. Performance of the two commercial check hybrids is indicated with an *open circle* and *open triangle* for CH1 and CH2, respectively. Traits are early vigor at stages V3, V4, and V6 (EV_V3, EV_V4, EV_V6), emergence (EME), female flowering (FF), early plant height at stages V3, V4, and V6 (PH_V3, PH_V4, PH_V6), final plant height (PH_final), ear height (EH), root lodging (RL), tillering (TILL), dry matter content (DMC), and total dry matter yield (TDMY).

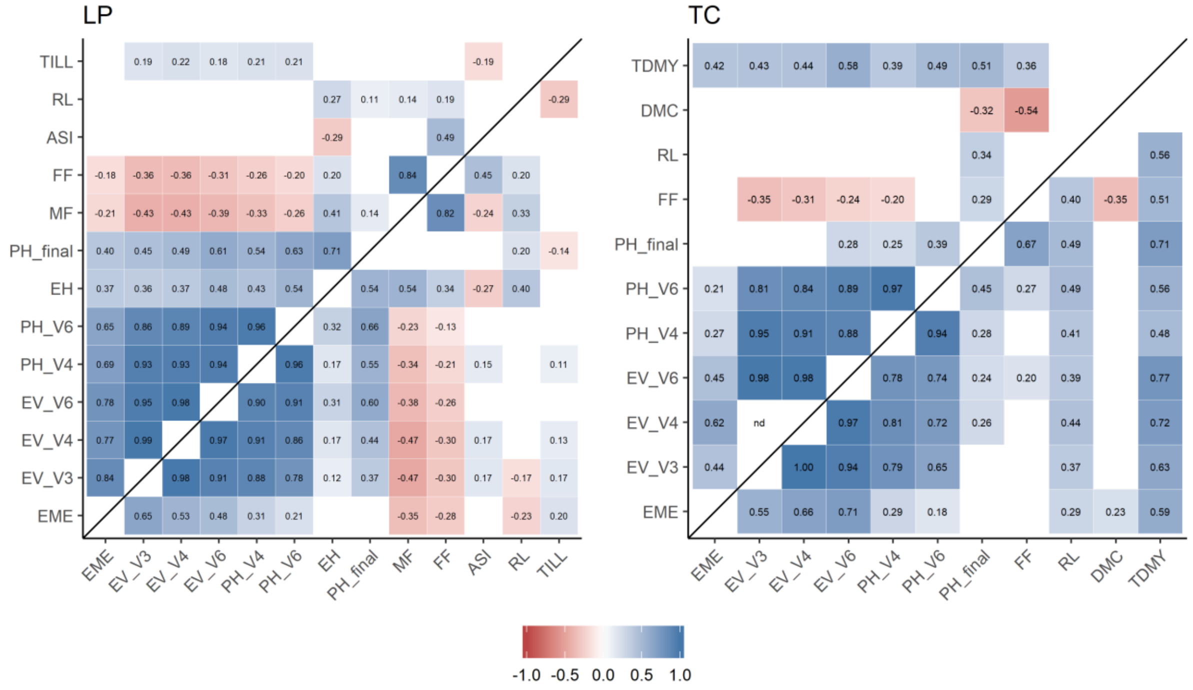

Fig. S3 Genetic correlation coefficients for line per se (LP, *left*, N = 471 (KE) and 402 (PE)) and testcross (TC, *right*, N = 190 (KE) and 188 (PE)) data within DH libraries KE (above diagonal) and PE (below diagonal) for traits emergence (EME), early vigor at stages V3, V4 and V6 (EV_V3, EV_V4, EV_V6), early plant height at stages V4 and V6 (PH_V4, PH_V6), ear height (EH), final plant height (PH_final), female flowering and male flowering (FF, MF), anthesis-silking interval (ASI), root lodging (RL), tillering (TILL), dry matter content (DMC, %), and total dry matter yield (TDMY, dt/ha). Values not exceeding twice their standard errors are blank, values labeled with "nd" did not reach convergence

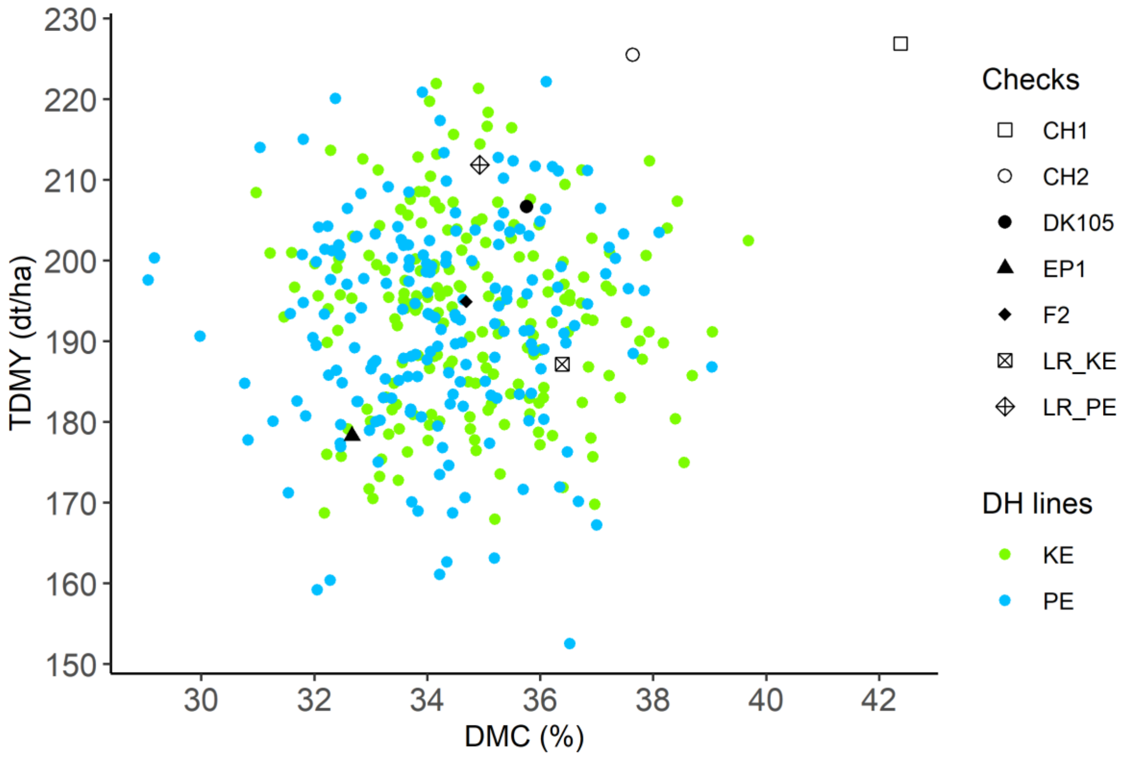

Fig. S4 Scatterplot of dry matter content (DMC) and total dry matter yield (TDMY) from testcross (TC) data of DH lines from landraces KE (N = 190) and PE (N = 188). Testcrosses of the landrace (LR_KE and LR_PE), lines used as checks (DK105, EP1, F2) and the performance of two commercial hybrids (CH1, CH2)

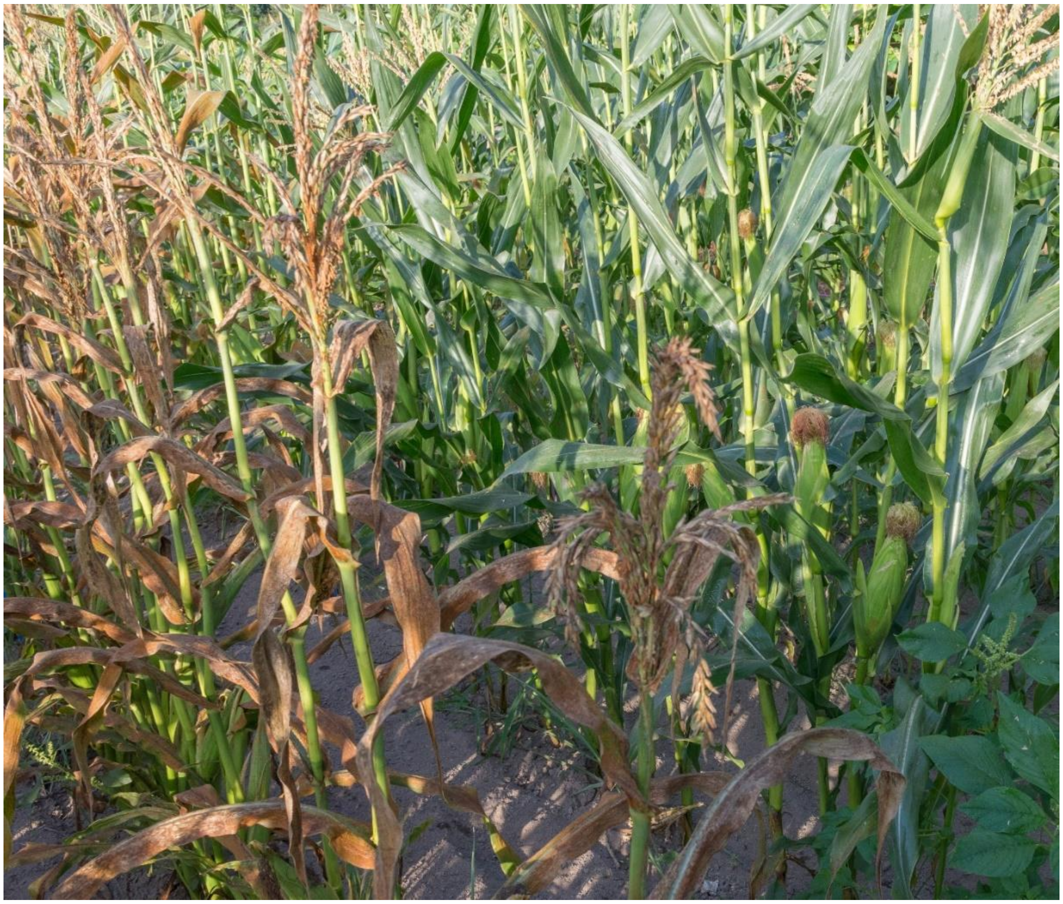

Fig. S5 Photograph showing the rust infection in line per se (LP) trials at location TOM in 2018. The genotype on the left was completely devastated by the infection, while the neighboring plots showed no symptoms at all

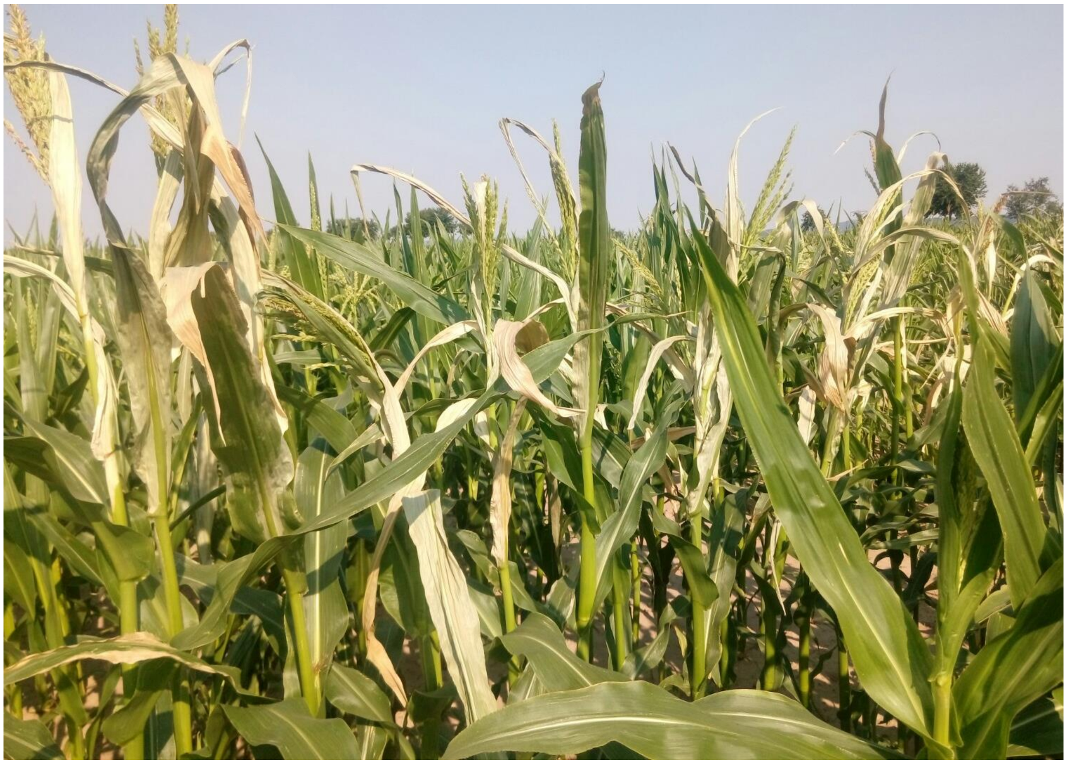

Fig. S6 Photograph showing the drought stress phenotype in line per se (LP) trials at location EIN in 2018. All plants of one genotype showed the same clear symptoms, while neighboring plots showed no damage
